# Review of Radiomics- and Dosiomics-based Predicting Models for Rectal Cancer

**DOI:** 10.3389/fonc.2022.913683

**Published:** 2022-08-09

**Authors:** Yun Qin, Li-Hua Zhu, Wei Zhao, Jun-Jie Wang, Hao Wang

**Affiliations:** ^1^ School of Physics, Beihang University, Beijing, China; ^2^ Department of Radiation Oncology, Peking University Third Hospital, Beijing, China; ^3^ Cancer Center, Peking University Third Hospital, Beijing, China

**Keywords:** radiomics, dosiomics, machine learning, deep learning, rectal cancer

## Abstract

By breaking the traditional medical image analysis framework, precision medicine–radiomics has attracted much attention in the past decade. The use of various mathematical algorithms offers radiomics the ability to extract vast amounts of detailed features from medical images for quantitative analysis and analyzes the confidential information related to the tumor in the image, which can establish valuable disease diagnosis and prognosis models to support personalized clinical decisions. This article summarizes the application of radiomics and dosiomics in radiation oncology. We focus on the application of radiomics in locally advanced rectal cancer and also summarize the latest research progress of dosiomics in radiation tumors to provide ideas for the treatment of future related diseases, especially ^125^I CT-guided radioactive seed implant brachytherapy.

## Introduction

According to the latest global cancer data for 2020 released by the International Agency for Research on Cancer of the World Health Organization, colorectal cancer ranks third and second in the global morbidity and mortality rates, respectively (1). In 2020, there were estimated 43,340 cases of rectal cancer diagnosed in the United States (2). Therefore, early detection of rectal cancer and precise treatment reducing the incidence, recurrence rate, and mortality rate of rectal cancer are very critical. There are many ways to treat rectal cancer, and in order to determine the best treatment regimen and optimize patient outcomes, efficient imaging biomarkers are needed to contribute to cancer detection, diagnosis, the choice of therapeutic strategy, prognosis inference, the prediction of response, and surveillance (3).

With the rapid development of imaging technology, medical imaging including computed tomography (CT), magnetic resonance imaging (MRI), or positron-emission tomography (PET) images play a significant role in clinical applications, particularly in cancer prognosis. Radiographic imaging technology mainly evaluates the grade of morphology of the tumor and its surrounding environment. However, it is difficult to convert the microheterogeneity and biological characteristics of the tumor into a quantitative mode (4). On the other hand, visual analysis is insufficient to capture the deep information of the lesions; thus, it cannot meet the requirement of accurate medicine and personalized treatment.

In recent years, advances in the use of artificial intelligence (AI) and computing methods in medical image processing and analysis transformed these images into quantitative data (5). Due to radiomics covering almost all solid tumors, it has been widely applied in oncology. Radiomics based on high-throughput feature extraction algorithms (manually defined and deep learning) enables the integration of imaging and clinical features to decode information hardly recognized by the naked eye and then use machine learning to model these proposed features, thus improving the efficiency of assessing prognosis and response. The current research model established through deep learning features has gradually been developed, but compared with huge radiomics features, this method-related research is still small (6–8).

The outcome of radiation therapy for tumors is closely related to dose distribution, but simple dose statistics alone cannot make accurate predictions about the outcome of radiation therapy. In response to this problem, the dosiomics approach can describe the dose distribution by the dose characteristics of intensity, texture, and shape, with higher accuracy, granularity, and spatial information, and is an effective method for parametric radiotherapy dose distribution (9). At present, there is still a gap in the application of dosiomics in rectal cancer.

Rectal cancer is the most common gastrointestinal malignant tumor in the world. More than 100,000 people are diagnosed with rectal cancer every year, 70% of which are locally advanced (T3–4 or N+) rectal cancer (LARC) (10). Unfortunately, the initial clinical symptoms of rectal cancer are not typical, and many patients are already in the locally advanced stage when they are first diagnosed, with estimated 149,500 new rectal cancer (RC) cases and 52,980 expected deaths in the United States in 2021 (11). In colorectal cancer, rectal cancer incidence is slightly higher than that of colon cancer. Rectal cancer has an insidious early onset, clinical symptoms are atypical, and many patients are already in the local progression stage when they got their first diagnoses. In this paper, we review the research status and progress of radiomics in the differential diagnosis, efficacy evaluation, and prognosis evaluation of LARC in recent years and look forward to the current research status of dosiomics and the future application of prognosis prediction of rectal cancer.

## Radiomics

**Figure 1:**
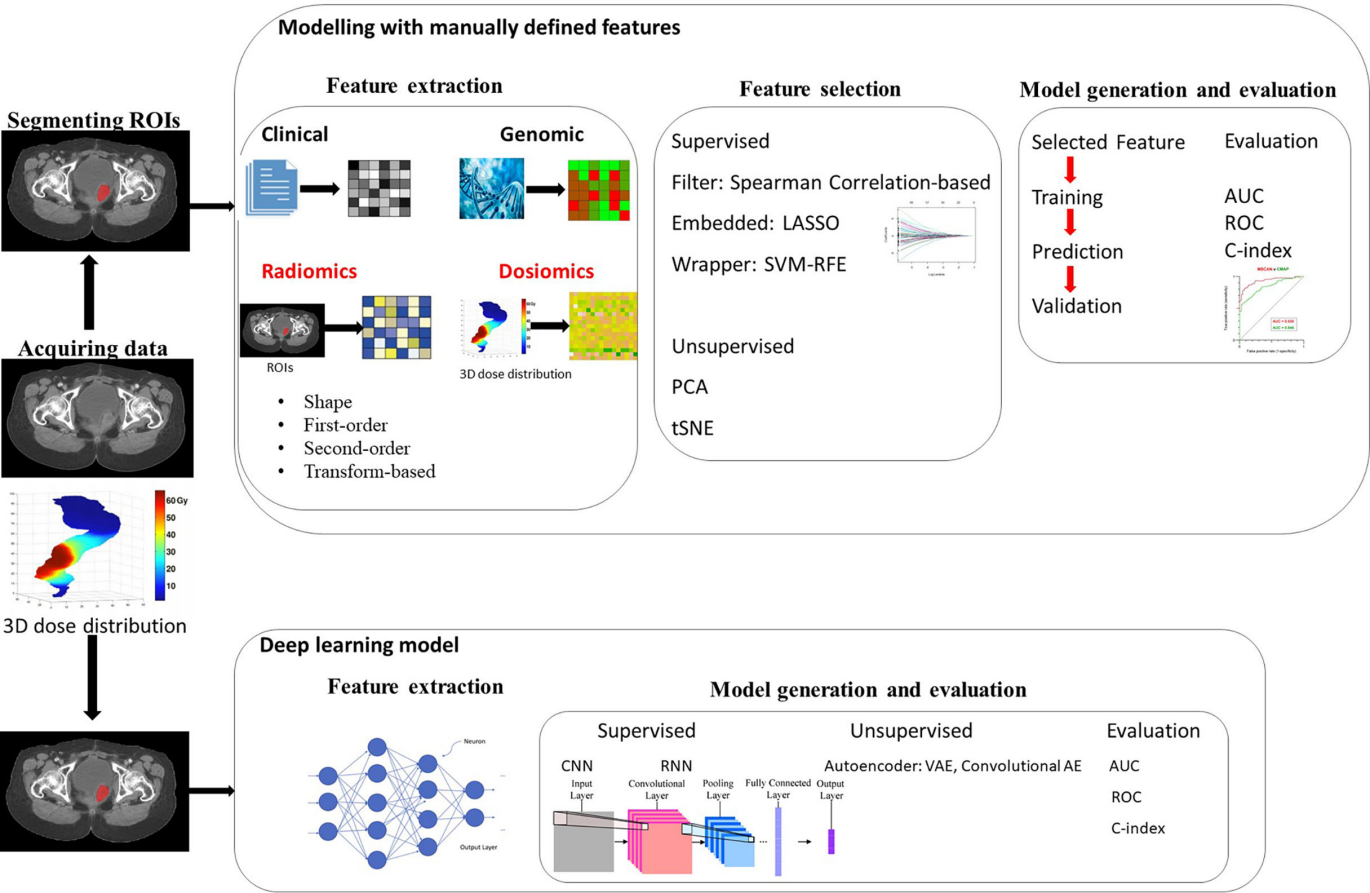
Workflow for radiomics and dosiomics analysis with feature-based (machine learning) and featureless (deep learning) approaches.

“Omic” generally refers to the mining of more parameters or features from the research target as a whole to further enrich the dimensions for research or reference. Similarly, radiomics refers to extracting quantitative information from morphological and functional imaging and is combined with clinical features, protein genome information, and identifying feature subsets related to prognosis through machine learning. Radiomics can be performed for both healthy human tissues and diseased tissues. It is a multidisciplinary and multi-imaging technology. Its primary analysis process is as follows (12): (1) identifying clinical problems; (2) access and process to high-quality standardized medical image data; (3) the segmentation of ROIs (regions of interest); (4) high-throughput radiomics feature extraction; (5) feature selection; and (6) prediction model establishment and statistical analysis. Radiomics enables the rich information in medical images to be fully displayed and provides deep quantitative features that cannot be recognized from visual inspection. Compared with traditional imaging methods, radiomics is a promising alternative, which can provide the basis for treatment plans, curative effects, and prognosis assessment for various diseases. Therefore, every radiomics step in the process is extremely of vital importance and needs to be performed rigorously. A workflow diagram illustrating the radiomics and dosiomics analysis process is shown in **Figure 1**.

### Identifying Clinical Problems

At present, various radiomics methods have been explored, aiming at realizing personalized medicine, such as the diagnosis (13–15), treatment response (16–18), and prognosis prediction (19, 20) of tumor cancers. At the stage of model construction, there are many similarities among diagnostic models, mainly faced with the selection of predictive factors (feature selection), the formulation of modeling strategies (algorithms), and the evaluation of the final model performance.

To solve clinical problems, the correlation between models and problems should be explored. Clinical problems determine the radiomic research direction and route, so different problems require different types of research design. Currently, the application of radiomics in rectal cancer is mainly the prognosis prediction of patients with LARC to neoadjuvant chemoradiotherapy (NCRT). Although the local recurrence (LR) rate of rectal cancer has been significantly reduced and the disease-free survival (DFS) rate has been significantly increased with the advances of clinical medicine and medical technologies, the LR of rectal cancer and the associated prognostic risk factors are still the major concerns for clinical rectal cancer treatments.

### Acquiring and Processing Data

Building an adequate database is the prerequisite for radiomics research. This is due to insufficient data capacity, which may reduce the model prediction accuracy and increases overfitting risk. The collected data are supposed to contain high-quality, standardized medical images and necessary clinical information, such as, pathological data and biological and genomic medical records. Commonly used medical images include CT, MRI, and PET/CT. Currently, rectal MRI is the preferred imaging modality for the local staging of rectal cancer. MRI is the superior imaging modality for the evaluation of primary tumor location, extension, and mesorectal fascia involvement (21) and is considered as the standard for the evaluation and staging of rectal cancer. For patients with local recurrent rectal cancer, most of the intraluminal recurrent tumors are diagnosed by rectal examination or direct visualization on rectosigmoidoscopy and MRI is the most accurate imaging method to detect and identify patients with extravascular recurrence (22), but its cost limits its application in routine follow-up; thus, usually, pelvic CT examination is performed after rectal cancer surgery. High-spatial-resolution T2 weighted imaging is the most important MRI sequence in the evaluation of rectal cancer and anatomic structures (23).

High quality and standardization of medical images is vital for feature extraction and quantification in radiomics. Ideally, the same scanning machine should be used as far as possible, and reasonable layer thickness, pixel size, tube voltage, and other parameters should be selected to obtain more ideal analysis results. However, this is not the case in many practical situations. Therefore, corrections should be considered in the subsequent analysis and modeling steps. Data preprocessing includes removing artifacts from the images, correcting inhomogeneity, intensity normalization, spatial smoothing, spatial resampling, noise reduction, and MRI field non-uniform registration (rigid, deformable, or mutual intensity algorithm) and reslicing, and so on (9, 24). Otherwise, the extracted features and the generated model will not be reproducible and non-generalizable.

The Image Biomarker Standardization Initiative (IBSI) has defined reporting guidelines that work toward standardizing the extraction of image biomarkers from acquired imaging to high-throughput quantitative image analysis (radiomics). Alex Zwanenburg et al. standardized 169 radiomics features to verify and calibrate different kinds of radiomics software, which will increase the reproducibility of radiomics studies and facilitate the clinical translation of radiomics (25).

### Segmenting Regions of Interest

The ROI segmentation uncertainty and time efficiency from the revised image data set are the most critical. There are two main areas of interest in the clinic: the tumor target area and the nearby organs at risk. At present, most researchers extract features from the gross tumor volume (GTV) to build models. The GTV is the position and extent of the primary rectal tumor. The clinical target volume (CTV) describes the extent of microscopic, unimageable tumor spread, and the planning target volume (PTV) allows for uncertainties in planning delivery. Additionally, the normal tissue structures in the vicinity of the target must be considered. The current methods used to segment ROI are mainly divided into automatic segmentation and manual segmentation. Although semi-automatic and fully automatic segmentation software have been widely used in radiomics research, especially for tumors with clear boundaries and regular morphology, the automatic segmentation method is relatively efficient and highly repeatable. It can meet the requirements of massive data segmentation (26). Of course, there are also studies based on the ROI sketched manually by doctors on the radiotherapy planning system. The advantage of manual segmentation is that the accuracy is high, but the results are easily affected by doctors’ subjective factors. For instance, interobserver delineation in cervical cancer can lead to significant differences and is reported to differ up to 4 cm (27). The intraclass correlation coefficient (ICC) can be used to reject non-reproducible features. For lesions where the boundary is not easy to detect, manual segmentation can be used; for tumors with clear boundaries and regular morphology, semi-automatic or automatic segmentation methods are efficient and highly reproducible, which can meet the requirements of massive data segmentation (26). Deep learning has been used to segment rectal tumors automatically. Weijun Chen et al. evaluated the results of two automatic contouring softwares (deep learning auto-segmentations and Atlas) on the OAR definition of CT images of lung cancer and rectal cancer patients (28). The results show that deep learning auto-segmentations were better than that of Atlas and can be used clinically. Hai-Tao Zhu et al. proposed a volumetric U-Net model that can automatically segment the rectal tumor region on the diffusion-weighted imaging images of LARC (29).

Accurate image segmentation ROI is the premise of radiomics analysis, and the segmentation algorithm with high accuracy and good repeatability still needs to be further studied. Noise, artifacts, and tumor infiltration to the surrounding normal tissues often conceal the lesion’s real edge, which brings great difficulty to image segmentation. The features extracted depend on the segmented area, rather than unclear or complex tumor boundaries, which can lead to inconsistent and low reproducibility of the results. Therefore, when different methods are selected for target area sketching (manual, semi-automatic, automatic sketching), the selection should be based on the required precision and time. At present, the commonly used segmentation method for many clearer tumor contours is through computer-assisted edge detection and then further manual adjustment. For patients with locally recurring rectal cancer (LRRC) treated with seed implantation, because the tumor structure has been interfered many times in previous treatments (including surgery, external radiotherapy, and seed implantation), the tumor margin merged with the normal tissue structure, resulting in the tumor border not being clear enough. Furthermore, these patients need to consider whether there are blood vessels, bones, or organs in the direction of the needles, so manual segmentation remains the gold standard.

### Feature Extraction

The core step of radiomics is to extract features from the ROI and then use them for quantitative analysis.

1. The manually extracted features mainly include 5 types: shape features (30), first-order statistics features or histogram-based features (31), second-order statistics features or textural features (32, 33), transform-based features (34), and some features that are obtained from PET images (SUV value) (35) and are only applicable to the fractal and fusion features of multi-mode images. Published studies are not only based on the radiomics features of PET but also based on the combined radiomics features of CT and MRI for the improvement of the accuracy for the rectal cancer prognostic model.
2. Deep learning features: the features extracted based on deep learning are different from handcrafted radiomics. It directly builds a deep learning model for the entire medical image. This method of extracting features requires a large data set. However, the database for treating LRRC with particle implantation is not enough. The extracted features are usually non-interpretable. At present, the deep learning of medical images usually utilizes convolutional neural networks (CNNs), which are neural networks with automatic feature extractors specially designed for images. Xception, VGG16, VGG19, ResNet50, InceptionV3, and Inception ResNetV2 are six commonly used CNNs (36). Deep learning is relatively new and has tremendous potential waiting to be explored (37).

The deep learning features are able to be combined with other relevant data, including necessary clinical information, pathological data, biological or genomics medical records, dosimetry, and so on to construct a robust model.

### Feature Selection

A feature is necessary to screen the imaging features acquired in ROI, make the established model universal, and avoid overfitting (38). Feature selection refers to the dimensionality reduction of many feature data extracted in ROI to obtain features related to the research endpoint.

In general, the methods of feature dimensionality reduction are divided into two categories: supervised and unsupervised. There are three supervised feature selection methods: filter, embedded, and wrapper methods (39).

(1) The filtering method is usually utilized as a preprocessing step, and feature selection is completely independent of any machine learning algorithm. It selects features based on the scores in various statistical tests and various indicators of correlation. Correlation filtering judges the correlation between features and tags. Commonly used methods include the mutual information method, chi-square filtering, F-test, Wilcoxon rank-sum test, the Fisher score, the Student’s t-test, and so on (40, 41). The relevance filtering method is a univariate feature selection method, meaning it does not consider the correlation between each feature.
(2) The embedding method is a method that allows the algorithm to decide which features to be used, that is, feature selection and algorithm training are performed at the same time. When using the embedding method, some machine learning algorithms and models are used for training to obtain the weight coefficients of each feature. Compared with the filtering method, the result of the embedding method will be more accurate to the model’s utility, which has a better effect on improving the model’s effectiveness. Using the embedding method, it is easy to achieve feature selection: reduce the amount of calculation and improve the model’s performance. Commonly used embedded methods are ridge regression, tree-based algorithms such as the random forest (RF) classifier, or the least absolute shrinkage and selection operator (40, 41).
(3) The wrapper method is a method of feature selection and algorithm training at the same time. The packaging method often uses an objective function to select the best feature subset instead of inputting a certain evaluation index or statistic threshold. The most typical objective function is the recursive feature elimination method, and some other wrapper methods include forward feature selection, backward feature elimination, exhaustive feature selection, bidirectional search feature selection, or bidirectional search (39, 40). The effect of the wrapper method is the most conducive to improve the model’s performance among all the feature selection methods. It can use very few features to achieve excellent results. In addition, when the number of features is the same, the performances of the packaging method and the embedding method are comparable, but it is faster than the embedding method, although its calculation amount is also very large, not suitable for too- large data.

Unsupervised feature selection methods are mainly principal component analysis (PCA) and cluster analysis. Each feature selection method has its strengths and weaknesses, and the performance depends on the type of the data set and the constraints related to the scenario.

### Model Generation and Evaluation

After feature extraction, a radiomics model must be established. Many studies use machine learning and deep learning methods to build prediction and classification models, which is currently the mainstream of published studies. Research endpoints usually include but are not limited to disease diagnosis (classification) and prognosis prediction [overall survival (OS), local control (LC), distant metastasis (DM), treatment response]. For example, for the disease of LARC, many studies have used radiomics to evaluate treatment response after neoadjuvant therapy. Other research endpoints are mainly DM, OS, and so on.

The machine learning algorithm models for predicting the treatment response of LARC after neoadjuvant radiotherapy and chemotherapy mainly include logistic regression, RF, and support vector machine (SVM) methods. To predict DM and the survival time of patients after radical resection for rectal cancer, Mou Li et al. used multivariate logistic regression (LR) analysis to establish a combined model of radiomic characteristics (Rad-score) and clinical factors (42).

The workflow of radiomics based on deep learning is essentially different from the workflow described above. Deep learning is based on representation learning in which the algorithm learns the best features to carry out a given task on its own by navigating the provided data (43). In deep learning–based radiomics, different network architectures, such as CNNs or autoencoders, are used to find the most relevant input data features. It does not need to define or select features in advance, but some studies are still related to statistics. Whereas radiomics captures quantitative values of shape and texture based on predefined mathematical terms, neural networks have recently been used to directly learn and identify predictive features from medical images (44). In addition, the process of data representation and prediction is carried out jointly (45). Of course, the features extracted by deep learning methods can be further analyzed and classified by the neural network, or they can leave the network and use different classifiers, such as decision trees, regression models, or support vector machines, to make predictions. If enough patients are included in the cohort, solutions leveraging deep learning should be the preferred method in the years to come (46).

Of course, the AI algorithm is not widely used in feature analysis. In many prognostic analysis cases, statistical methods such as Spearman correlation and univariate and multivariate Cox regression or most machine learning methods (logistic regression) are used for analysis. However, many studies have shown that the predictive performance of AI models is superior to traditional statistics. For example, Quirino Lai et al. conducted a systematic review of the role of AI in the prognosis of liver cancer patients and concluded that AI has a perfect role in clinical research and the application of hepatocellular carcinoma (HCC) (47). The accuracy of the prediction model is related to the sample size and feature parameter selection and also to machine learning algorithms.

### Delta-radiomics

There are usually two types of extracted features. One is the single-time-point radiomics, where features are extracted from a particular image (e.g., pretreatment), and the other is delta-radiomics (48). The delta-radiomics feature is defined as the difference in radiomics feature before and after a specific treatment method. They can be calculated between the pretreatment and post-treatment features. With the changes of radiomic features over time in longitudinal images, delta radiomics can potentially be used as a biomarker to predict treatment response and offer abundant information to identify, quantify, and potentially predict therapy-induced changes throughout treatment (49). Radiomics features extracted only before the treatment cannot reflect the overall treatment details, while delta-radiomics can describe the changes in the image during the process, which is relatively more rigorous. The process of delta-radiomics and radiomic research is the same. After extracting the delta-radiomics features from the original ROI, due to the large number of radiomic features extracted from the images, many methods mentioned above are used to rule out redundant delta-radiomic features (DRFs). The selected DRFs are then tested to determine their significance as a treatment response function using linear regression models, t-test, and mixed-effect models (50). Significant DRFs are further tested and modeled using machine-learning algorithms to create a model that can predict the outcome of a new patient.

Some studies investigate the effectiveness of delta-radiomics compared to single-time-point radiomics, and the results indicated that delta features could provide better treatment assessment than single-time-point features. Studies on delta-radiomics have been reported since 2017. These articles have all been published in the last 5 years and are gradually increasing every year. Among the 54 articles screened, a total of 7 (13%) studies reported on the use of delta-radiomics in rectal cancer. A total of 10 (19%) studies referring to the use of delta-radiomics in the prediction among patients with lung cancer were excluded in this review. Other studies using delta-radiomics in grade osteosarcoma and pancreatic and gastric cancer were also excluded. Some studies have shown that delta-radiomics can successfully predict the prognostic response of rectal cancer, including the complete pathological response (pCR), DM, LR, and DFS (51–56). For instance, Seung Hyuck Jeon et al. developed delta-radiomics signatures to predict treatment outcomes after preoperative chemoradiotherapy and surgery in LARC (52). Giuditta Chiloiro et al. used delta-radiomics to investigate the correlation between changes in magnetic resonance imaging (MRI) radiomic characteristics before and after neoadjuvant radiotherapy (NCRT) LARC patients and the 2-year DM rate (53).

## Radiomics in rectal cancer

Radiomics, based on advanced pattern recognition tools, has been widely studied for clinical prediction models in diagnosis and treatment prognosis/selection in oncology. In recent years, radiomics has been gradually applied to histopathological grading (57–59), pretreatment staging prediction, differential diagnosis, efficacy evaluation, and prognosis evaluation for rectal cancer. Many studies have shown that radiomic features can objectively provide texture information related to histopathological and immunohistochemical markers and can non-invasively evaluate biological characteristics such as tumor proliferation, migration, and angiogenesis before treatment. This section will discuss an overview of notable studies about LARC published in this area.

### Radiomics Prediction of Locally Advanced Rectal Cancer After Neoadjuvant Chemoradiotherapy

NCRT can reduce the tumor size and recurrence and increase the tumor resection rate and anus retention rate with a very slight side effect (60). Therefore, it is very necessary to refine the selection of appropriate patients and irradiation mode of NCRT. Since most patients with rectal cancer have locally advanced diseases at the time of diagnosis, NCRT is the standard recommendation to improve the prognosis of patients.

However, in the context of precision medicine, it is an urgent problem in clinical work to find a method that can predict the therapeutic effect early and avoid the therapeutic risk, effectively guide the individual treatment, and improve the prognosis of patients to the greatest extent. Therefore, it is crucial to screen LARC patients with therapeutic responses to NCRT. The evaluation of treatment response to NCRT is still challenging. The complete pathologic response (pCR) to NCRT is assessed during the pathological examination after surgery. Identifying patients in pCR with a high accuracy rate could lead to improved clinical outcome (61). Radiomics also has important clinical significance in evaluating NCRT efficacy and the prognosis of rectal cancer. The most commonly used medical images are CT and MRI. In recent years, many studies have predicted and verified the efficacy of NCRT in LARC through radiomics.

According to the WHO, the solid tumor efficacy evaluation criteria are divided into complete remission (complete response, CR): tumor disappears completely, lasting more than 4 weeks; partial remission (partial response, PR): tumor shrinkage ≥50%, lasting more than 4 weeks; stable (stable disease, SD): tumor enlargement <25%, shrinkage <50%; progress [progressive disease (PD)]: tumor increases by more than 25% (62). PR and SD are considered effective. PD means that the treatment is ineffective. Whether a patient has achieved pCR is often determined by postoperative pathological examination, and it is not known whether it is remission before surgery. Many recent studies have found that radiomics can help clinicians predict whether patients will be pCR after NCRT before surgery to avoid excessive treatment and the burden and pain caused by surgery and improve treatment accuracy (61–73). CT-based radiomics has shown promise in LARC. The following table study endpoint is from the four aspects: pCR DFS, downstaging, and distant control. **Table 1** shows the studies of LARC radiomics using CT images in the PubMed database.

**Table 1:** Summaries of selected locally advanced rectal cancer (LARC) radiomics studies (CT).

**Conclusion**	Entropy, uniformity, and standard deviation were independent texture features in predicting DFS	Low- and high-risk groups for DFS in the training set ([HR]56.83; P < 0.001) in the validation set (HR52.92; P < 0.001).	The DNN predicted complete response with an 80% accuracy.	Radscore ([OR] = 13.25; [95% CI],4.06–71.64; p < 0.001) Age (OR = 1.10/1 year; 1.03–1.20; p = 0.008)	OS from 0.672 [0.617 0.728] with clinical features only to 0.730 0.658 0.801]	83.9% accuracy in predicting TRG 0 vs. TRG 1–3 in validation.	AUC=0.842 (training set) AUC=0.802 (validation set)
**Feature selection model**	LoG spatial filter	ICC LASSO Cox model	Wilcoxon test, p<0.05 ICC	Penalized logistic regression	Spearman correlation coefficient	Keep high ROC	LASSO
**Statistical method**	independent t-test log-rank test MCPHM	Chi-square log-rank tests	DNN SVM LR	Univariable analysis MLR	Unsupervised and supervised method	LOR RF SVM	MLR
**Imaging modality**	CT	CT	CT	CT	CT	CT	CT
**Number of patients**	95	108	95	121	411	91	148
**Study endpoint**	Response, DFS	DFS	PCR	Downstaging	LC, DFS ,OS Distant control	PR	Distant metastases, OS
**Accepted**	August 10, 2017	January 22, 2018	August 3, 2018	March 11, 2019	October 25, 2019	April 6, 2020	August 13, 2020
**Author**	Chee et al.	Yankai Meng, et al.	Jean et al.	Ben et al.	Jiazhou et al.	Zhigang et al.	Mou Li, et al.

ICC, intraclass correlation coefficient; DNN, deep neural network; SVM, support vector machine; LIR, linear regression; LOR, logistic regression; RF, random forest; HR, hazard ratio; OR, odds ratio MLR, multivariate logistic regression; MCPHM, multivariable Cox proportional hazards model; LoG, Laplacian of Gaussian; ROC, receiver operating characteristic.

An overview of the main studies proposing MRI radiomics for the pCR of NCRT prediction outcomes is reported in **Table 2**. In all studies, three-dimensional (3D) manual segmentation of the primary tumor was performed to extract radiomic features and the performance of the models were calculated by receiver operating characteristic curves.

**Table 2:** Summaries of selected LARC radiomics studies (MRI).

**Validation**	Internal validation (4-fold validation)	Internal validation (train/test split)	Internal validation (train/test split)	Internal validation (train/test split)	Internal validation (train/test split)	Internal validation (4-fold validation)	Internal validation (train/test split)	Internal validation (train/test split)	Internal validation (train/test split)	External validation
**Performance**	AUC =0.84 for pCR AUC =0.89 for GR	AUC = 0.9756 (training) AUC = 0.9799 (test)	AUC=0.93 [95% CI: 0.84, 1]	AUC= 0.948 [95% CI, 0.9070.989] AUC= 0.966 [95%CI, 0.924-1.000]	AUC = 0.908, 0.902, 0.930	Handcrafted features AUC: 0.64 DL-based features AUC: of 0.73	AUC =0.75 (training) AUC = 0.75 (test)	Training cohort AUC =0.94 (95% CI: 0.82–0.99) validation cohort AUC=0.80 (95% CI: 0.58–0.94)	AUC = 0.84,0.88 (training) AUC = 0.81,0.75 (test)	Training cohort AUC= 0·868 [95%CI 0·825–0·912] validation cohort 1 AUC=0·860 [95%CI 0·828–0·892] validation cohort 2 AUC=0·872 [95%CI 0·810–0·934]
**Model**	Artificial neural network	Logistic regression	Random forest classifier	Radiomics nomogram	SVM	LASSO-logistic regression models	SVM	LASSO logistic regression	Logistic regression	RAPIDS prediction signature
**ROI software**	Manual segmentation	Manual segmentation	Manual segmentation	Manual segmentation	Manual segmentation	Manual segmentation	Manual segmentation	Manual segmentation	Manual segmentation	Manual segmentation
**Number of** **patients**	48	222	114	186	134	43	102	67	165	933
**MRI Imaging modality**	Pre- CRT MRI (T1\T2WI, DWI, and DCE)	Pre- and after CRT MRI (T2WI and DWI )	After CRT MRI (T2WI)	Pre-CRT MRI (T1\T2WI, DWI, and ADC)	Preoperative MRI (T2WI)	MRI (DWI and ADC map)	Pre- CRT MRI (T2WI)	Pre- CRT MRI (T2WI)	Pre- and after CRT MRI (T2WI and DWI )	Pre- CRT MRI (T1\T2WI and DWI)
**Study endpoint**	PCR GR	PCR	PCR	PCR	PCR, GR downstaging	PCR	PCR	Non-responders	PCR	PCR
**Accepted time**	May 19, 2016	September 22, 2017	January 2, 2018	July 27, 2018	June 6, 2019	February 24, 2020	April 15, 2020	May 14, 2020	October 20, 2020	January 4, 2022
Author	Ke Nie, et al.	Zhenyu Liu, et al.	Natally, et al.	Yanfen Cui, et al.	Xiaoping Yi, et al.	Jie Fu, et al.	Iva Petkovska, et al.	Bianca Petresc, et al.	Lijuan Wan,et al.	Lili Feng, et al.

PCR, pathological complete response; GR, good response; AUC, area under the curve; CI, confidence interval; ROI, region of interest; T1WI, T1-weighted imaging; DWI, diffusion-weighted imaging; T2WI, T2-weighted imaging; DCE, dynamic contrast enhanced.

It was concluded that the features of MRI prior to treatment could effectively predict patients who were unresponsive to NCRT. Therefore, MRI−based radiomics has important clinical significance in the NCRT efficacy evaluation and prognosis evaluation of rectal cancer. This could provide an improved basis for personalized treatment. Certainly, radiomics has a predictive value for the NCRT curative effect of LARC and shows good predictive value in terms of tumor staging, postoperative metastasis, and prognosis after treatment.

## Development and challenges of locally recurrent rectal cancer

Despite advances in surgical techniques and chemoradiation therapy, recurrent rectal cancer remains a cause of morbidity and mortality (74). LRRC is the recurrence of a tumor of the same pathologic nature in the primary tumor after surgery, in the pelvis, in the field of operation. Neoadjuvant therapy and surgical treatment for rectal cancer have been improved and the concept of comprehensive treatment has been promoted, and the survival of patients with rectal cancer has been improved. However, rectal cancer recurrence remains a common clinical problem, and patients generally have a dismal prognosis and a poor quality of life.

LRRC has various treatment methods, including surgery, external beam radiotherapy, intraoperative radiotherapy, Iodine-125 (I-125) seed implantation, heat therapy, and radiofrequency ablation (75). For patients with postoperative recurrence, due to the damage to the normal anatomical structure and the adhesion of recurrent lesions and surrounding tissues, the reoperation resection rate is low. Therefore, the treatment means and effect are not satisfactory. For those who have received external beam radiotherapy, it is difficult to improve the treatment effect due to increasing the local dose. For patients with recurrent rectal cancer after surgical resection or external radiotherapy, LRRC prognosis is poor, while CT-guided ^125^I seed implantation therapy has become a recommended therapy. The I-125 seed Model 6711 consists of a titanium cylindrical tube with 0.8-mm radius and 4.5-mm length, with an average energy of 28 Kev and a half-life of 59.4 days is commonly used throughout oncology centers worldwide. A dose prescription of 110–160 Gy was considered, with an initial source activity of 0.4–0.7 mCi. The particle spacing is usually 1 cm, which is easier to identify particles. Therefore, permanent 125I seed interstitial brachytherapy is a potential salvage modality because of its unique physical and clinical characteristics. The 2016 National Comprehensive Cancer Network Clinical Practice Guidelines in Oncology has recommended radioactive 125I seed (RIS) implantation for the treatment of LRRC (76).

Many factors affect survival, following the treatment of LRRC. We can also consider the background liver condition, the radiologic and histologic characteristics of the tumor, biologic markers, and comorbidities. Traditionally, conventional linear models, such as the survival analysis and the Cox proportional hazard models, have been used to evaluate LRRC prognosis. Nevertheless, linear systems can have considerable limitations and often fail to capture the complexity of clinicopathological characteristics. Therefore, it is very necessary to analyze the prognosis of LRRC, and there is still a gap in the application of radiomics in the prognosis of LRRC.

## Dosiomics and its application

Inspired by radiomics, the concept of dosiomics was formally proposed in 2017. It builds a radiotherapy result prediction model by extracting the characteristics of dose distribution, thus guiding the formulation of personalized radiotherapy plans. The patients’ 3D dose distributions can be considered as images with spatial and statistical distributions of dose levels. For radiotherapy for cancer, parameters such as the prescription dose, dose distribution, and dose–volume histogram (DVH) can also be used to assess the treatment response and prognostic analysis of cancer. Dosimetry texture features include: volume, dose, variance, center point position, contour boundary, spatially weighted DVH skewness, and kurtosis (77). Combining the characteristics of radiology and dosimetry can obtain more comprehensive information related to tumor radiotherapy, which helps to improve the accuracy of prediction. Unfortunately, there have been only 13 studies on dosiomics from 2018 to 2022, and there is no research on dosiomics in rectal cancer.

Linda Rossi et al. in 2018, applied dosimetric texture analysis (TA) features and DVH parameters to improve the prediction modeling of treatment complication rates in prostate cancer radiotherapy (78). Dosimetric texture analysis features characterizes the grayscale distribution in a patient’s 3D dose distribution image and derives image features to improve the features of the predictive model. The main dosimetric texture analysis features extracted by Linda Rossi et al. are the gray-level frequency histogram, gray-level co-occurrence matrix (GLCM), gray-level run length matrix (GLRLM), gray- level size zone matrix (GLSZM), and neighborhood gray tone difference matrix (NGTDM). Bin Liang et al. extracted the spatial characteristics of dose distribution in the ipsilateral, contralateral, and whole lung by dosimetry and then used them to construct a prediction model by single-factor and multifactor LR (79). The results showed that the spatial characteristics of dose distribution extracted by dosimetry effectively improved the predictive ability. Aiqian Wua et al. investigated whether dosiomics can predict an IMRT-treated patient’s locoregional recurrences (LRs) and get a comprehensive dosimetry and radiomics model can successfully divide patients into high- and low-risk groups(log-rank test, p=0.025), but the radiomics model alone cannot get same result (80). Furthermore, Lee et al. proposed a multiperspective data analysis method to predict weight loss in the acute phase of radiotherapy for lung cancer using radiomics and dosimetry texture features. In short, many studies on tumor radiotherapy are more effective in predicting models established by combining radiomics, dosimetry, and clinical data (81). Jin et al. extracted 42 radiomic features from CT images of 94 patients with esophageal cancer and combined them with 18 dosimetry parameters to predict the patient’s response to radiotherapy; the study results showed that the radiomic features were combined with dosimetry parameters. The subsequent AUC can reach 0.71, while the AUC using only radiomics is 0.69 (82). So far, the research on dosiomics in rectal cancer is vacant.

The authors found that found that compared with the predictive model established by radiomics alone, the model established by integrating the characteristic parameters of radiomics and radiotherapy dosimetry can effectively improve the predictive evaluation of tumors after radiotherapy. However, relatively few studies combine radiomics and dosiomics, and the application of dosiomic characteristics to predict the efficacy of radiotherapy is still in its initial stage. Therefore, the role of dosiomic characteristics in radiation oncology should be further studied.

## Limitations of radiomics

When applying radiomics research results to clinical practice, there are still some limitations and challenges in some aspects, mainly including the following:

**1.**Most of the previous studies were conducted retrospective analysis, and most of the included data came from the same institution. Therefore, a large sample and multicenter prospective research test are essential. It requires extensive cooperation in multiple disciplines and fields and is also an essential part of applying radiomics to clinical practice.
**2.**The reproducibility and repeatability of radiomics features still need to be discussed. This issue depends on the used imaging modality, sequence, scanning parameters, reconstruction algorithm spatial resolution, size of the image, image quality, reconstruction and correction parameters, and motion artifacts, and software used to extract radiomic features (83). The IBSI proposes the computed features of different institutions on a common data set. To expand this effort, a review proposes to include benchmarking data sets collected by different institutions to guarantee the maximum heterogeneity in terms of the acquisition parameters and develop an infrastructure, based on workflow programming language, that allows users to connect to the mentioned repository and run their feature extraction software (84). In terms of repeatability and reproducibility, deep learning-based radiomics may be advantageous. The self-learning neural networks show a better capability for generalization (85).
**3.**There is no theoretical basis to explain the biological meaning of radiomic features, which also hinders the further development of radiomics. Many models have been created and published, but these studies often lack standardized evaluation on external cohorts of patients, which also explains why not a single model has been translated to clinical practice. Published Radiomics quality score (RQS) and Transparent Reporting of a multivariable prediction model forindividual prognosis or diagnosis (TRIPOD) guidelines improve the validity of radiomics as a clinically accepted field. We need to overcome the challenges before radiomics can be successfully introduced into clinical settings. Efforts are being made to overcome these limitations

## Conclusions

With the rapid development of AI technology, radiomics based on machine learning and deep learning has broad application prospects. In the current background of advocating precision medicine and personalized treatment, in evaluating the efficacy of NCRT in LARC patients, a non-invasive, efficient, and accurate imaging omics prediction model is established for clinical application, and it has developed into a clinically useful model.

In summary, radiomics is an emerging diagnostic imaging technology that plays an essential role in predicting the effect of NCRT on LARC and can optimize treatment plans through process management, thereby improving the short-term and long-term prognosis of patients. This review also emphasizes the necessity of applying radiomics and dosiomics to the prognostic model of LRRC. In short, the application of radiomics and dosimetry in radiation oncology is of great value for doctors’ clinical decision-making, treatment planning, and follow-up workflow. However, published retrospective studies presented their own model with a certain degree of heterogeneity and do not facilitate translation into clinical practice. Therefore, the study design needs to be further improved, and the promotion and validation of prediction models need to be further explored, so as to strengthen the clinical application of radiomics.

## Future direction

With the continuous advancement in AI, radiomic and dosiomic diagnosis and prediction methods based on deep machine learning are the effective way to develop clinical research in the future. Drawing on the application of radiomics and dosiomics in other cancers, in terms of evaluating the efficacy of LRRC patients through seed implantation and second-course radiotherapy, establishes a fast, efficient, and accurate radiomics prediction model for clinical application. Furthermore, establishing additional predictive tools that can be used in clinics in a true sense, especially in the current background of advocating precision medicine and personalized treatment, will become a low-cost, non-invasive, and convenient new diagnostic evaluation method, thereby improving the prognosis of patients.

## Author Contributions

YQ performed the literature searches and contributed to draft versions of the manuscript. WZ performed the searches and revised the final version of the manuscript. LZ provided support on all aspects of the review and reviewed the manuscript. JW improved ideas for the writing of articles. HW provided support on all aspects of the review and reviewed the manuscript. All authors contributed to the article and approved the submitted version.

## Funding

This work is supported by the Innovation & Transfer Fund of Peking University Third Hospital (Grant No. BYSYZHKC2021113), Bei jing Lianying Intelligent Imaging Technology Research Institute Hospital-enterprise Joint Research and Development Platform Fund, No.H79462-07 and National Nature Science Foundation of China under Grants No. U1867210 and No.12175012.

## Conflict of Interest

The authors declare that the research was conducted in the absence of any commercial or financial relationships that could be construed as a potential conflict of interest.

## Publisher’s Note

All claims expressed in this article are solely those of the authors and do not necessarily represent those of their affiliated organizations, or those of the publisher, the editors and the reviewers. Any product that may be evaluated in this article, or claim that may be made by its manufacturer, is not guaranteed or endorsed by the publisher.
